# Molecular Crowding Tunes Material States of Ribonucleoprotein Condensates

**DOI:** 10.3390/biom9020071

**Published:** 2019-02-19

**Authors:** Taranpreet Kaur, Ibraheem Alshareedah, Wei Wang, Jason Ngo, Mahdi Muhammad Moosa, Priya R. Banerjee

**Affiliations:** 1 Department of Physics, University at Buffalo, SUNY, NY 14260, USA; tkaur2@buffalo.edu (T.K.); ialshare@buffalo.edu (I.A.); wwang23@buffalo.edu (W.W.); jasonngo@buffalo.edu (J.N.); 2 Department of Pharmacology and Chemical Biology, Baylor College of Medicine, Houston, TX 77030, USA; mahdi.moosa@gmail.com

**Keywords:** membraneless organelles, optical tweezer, liquid–liquid phase separation, protein diffusion, depletion interaction, entropic force, low-complexity sequences, intrinsically disordered proteins

## Abstract

Ribonucleoprotein (RNP) granules are membraneless liquid condensates that dynamically form, dissolve, and mature into a gel-like state in response to a changing cellular environment. RNP condensation is largely governed by promiscuous attractive inter-chain interactions mediated by low-complexity domains (LCDs). Using an archetypal disordered RNP, fused in sarcoma (FUS), here we study how molecular crowding impacts the RNP liquid condensation. We observe that the liquid–liquid coexistence boundary of FUS is lowered by polymer crowders, consistent with an excluded volume model. With increasing bulk crowder concentration, the RNP partition increases and the diffusion rate decreases in the condensed phase. Furthermore, we show that RNP condensates undergo substantial hardening wherein protein-dense droplets transition from viscous fluid to viscoelastic gel-like states in a crowder concentration-dependent manner. Utilizing two distinct LCDs that broadly represent commonly occurring sequence motifs driving RNP phase transitions, we reveal that the impact of crowding is largely independent of LCD charge and sequence patterns. These results are consistent with a thermodynamic model of crowder-mediated depletion interaction, which suggests that inter-RNP attraction is enhanced by molecular crowding. The depletion force is likely to play a key role in tuning the physical properties of RNP condensates within the crowded cellular space.

## 1. Introduction 

Ribonucleoprotein (RNP) granules or particles are a diverse group of subcellular compartments that are utilized by eukaryotic cells to spatiotemporally organize various biomolecular processes. These non-membranous assemblies, also termed as membraneless organelles (MLOs), dynamically form, dissolve, and tune their physicochemical microenvironment in response to changing cellular cues [1,2,3]. RNP granules are enriched in proteins with low-complexity domains (LCDs) that are structurally disordered [4,5,6], and are assumed to be formed by RNP liquid–liquid phase separation (LLPS) [7]. LLPS is a spontaneous physical process that results in the formation of co-existing liquid phases of varying densities from a homogeneous solution [2,8]. At the molecular level, low-affinity multivalent interactions amongst different LCDs and their partner nucleic acids provide the necessary energetic input to drive the LLPS of RNPs [9]. Furthermore, LCD-mediated promiscuous interactions can act synergistically with sequence-specific interactions in many RNPs, thereby shaping their global phase behavior [10]. Experimentally, it was observed that these promiscuous interactions are tuned by several cellular physicochemical perturbations (e.g., pH, salt concentration, and non-specific interactions with biomacromolecules) [10,11,12,13,14]. 

Unlike in typically utilized in vitro experimental conditions, in cellulo environments are crowded by a plethora of macromolecules that are ubiquitous within the cellular milieu [15]. To effectively capture biomolecular dynamics in a crowded cellular environment, in vitro studies utilizing recombinant proteins employ buffer systems containing inert biocompatible polymers as crowding agents. One of the most widely used crowders is polyethylene glycol (PEG), a neutral hydrophilic polymer with numerous applications in crystallography, biotechnology, and medicine [16,17,18,19]. Macromolecular crowding by PEG and similar polymer agents imparts a significant excluded volume effect (i.e., a space occupied by one molecule cannot be accessed by another) and results in alterations of molecular and mesoscale properties of biomolecules. For example, molecular crowding was shown to affect protein conformation [20,21,22,23], RNA folding [24], conformational dynamics of intrinsically disordered proteins [25], energetics of protein self-association [26,27,28], molecular recognition [29], and LLPS of globular proteins [30,31,32,33]. However, how macromolecular crowding alters disordered RNP condensation remains underexplored. 

The well-established excluded-volume model of colloid–polymer mixtures predicts that the addition of a polymer chain to a neutral colloidal suspension will trigger inter-colloid attraction [34]. The underlying driving force, known as the depletion interaction, is originated due to a net entropy gain by the system via maximizing the free volume available to the polymer chains. For globular protein–crowder mixtures, this depletion interaction can induce various phase transition processes, including protein crystallization [35]. In a similar vein, for RNP systems containing low-complexity “sticky” domains, a considerable impact of macromolecular crowding on their phase transition is expected [36]. This idea is supported by multiple recent observations such as (i) crowding induces homotypic LLPS of the nucleolar phosphoprotein Npm1 in vitro [37], (ii) PEG induces a robust liquid phase transition of the Alzheimer’s disease-linked protein Tau [38], and (iii) macromolecular crowding results in a substantial decrease in protein concentration required for inducing hnRNPA1 phase separation [39,40]. However, it remains unknown whether molecular crowding impacts the fluid dynamics of RNP condensates.

The material properties of intracellular RNP granules are important determinants of their function in intracellular storage and signaling [1,41]. Notably, many RNP condensates are competent to mature into a fiber-like state, which is implicated in several neurological diseases [11,42,43,44,45]. While the roles of LCD sequence compositions and charge patterns in controlling mesoscale dynamics of the RNP condensates have been the subject of some recent investigations [46,47], little is known regarding the effect of generalized thermodynamic forces such as crowding on RNP condensation. Here we conduct an experimental study to evaluate the impact of macromolecular crowding on the RNP liquid–liquid coexistence boundary, condensate fluidity in the micron-scale, and transport property by RNP diffusion in the nano-scale. Utilizing an archetypal RNP, fused in sarcoma (FUS), as well as representatives of the two commonly occurring LCD sequences in eukaryotic RNPs, we demonstrate an important role of crowding in modulating the mesoscale fluid dynamics of RNP condensates. 

## 2. Results

### 2.1. Macromolecular Crowding Facilitates FUS Condensation and Alters Droplet Fluid Properties 

**Figure 1:**
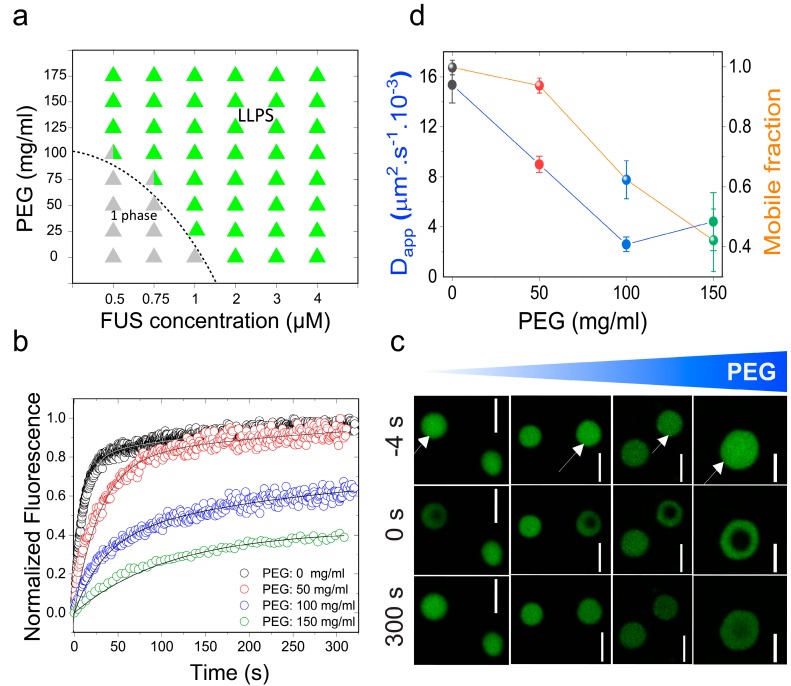
Molecular crowding facilitates full-length fused in sarcoma (FUS^FL^) liquid–liquid phase separation (LLPS) and tunes protein droplet viscoelastic properties. (**a**) Isothermal phase diagram of FUS-polyethylene glycol (PEG) 8000 mixtures. The dotted line indicates the liquid–liquid phase boundary. (**b**) Fluorescence recovery after photobleaching (FRAP) plots of FUS (10 μM) condensates at various concentrations of PEG8000. (**c**) Confocal fluorescence microscopy images corresponding to the data in Figure 1b are shown. Scale bar = 4 μm. Negative time implies droplets before bleaching. (**d**) An analysis of these FRAP results on the basis of a diffusion model reveals a scaling of apparent diffusion coefficients (D_app_; left axis, blue line) and the fraction of the mobile phase (right axis, orange line) with increasing concentration of PEG8000.

Initially, we chose to study the effects of molecular crowding on the phase behavior of FUS using PEG8000 at concentrations that mimic cellular macromolecular density (≥150 mg/mL) [48]. FUS is a stress-granule-associated RNP that undergoes LLPS in vitro and in cellulo via attractive inter-protein interactions [49,50]. Persistent FUS condensates also mature into a solid-like state during aging that is augmented by amyotrophic lateral sclerosis (ALS)-linked mutations [42,51]. Therefore, FUS serves as an ideal model system for this study. Firstly, we tested the impact of crowding on the liquid–liquid coexistence boundary of the full-length FUS (FUS^FL^) in a physiologically-relevant buffer (25 mM Tris, 150 mM NaCl; pH 7.5) with variable PEG8000 concentrations. We used solution turbidity measurements in conjunction with optical microscopy to construct a phase diagram of FUS^FL^–PEG8000 mixtures, which is presented in Figure 1a. We observed that FUS^FL^ formed micron-scale phase-separated droplets in vitro at protein concentrations ≥2 μM without any crowding agents (Figure 1), consistent with the protein’s ability to undergo LLPS at physiologically relevant concentrations [47,51]. Increasing PEG8000 concentration in the buffer solution from 0 to 150 mg/mL decreased FUS^FL^ phase separation concentration to <1 μM (Figure 1a). These results suggest that crowding by PEG8000 facilitates FUS^FL^ condensation.

The observed effect of PEG on lowering the RNP liquid–liquid coexistence boundary can be explained by a crowder-mediated depletion force that effectively increases the net inter-RNP attractive interaction with increasing concentrations of the crowder [52] (also see Note S1). If that is the case, we expect that the fluidity of RNP droplets will decrease with increasing macromolecular crowding due to the enhanced inter-molecular network strength within the condensed phase [41]. A similar phenomenon is known to drive colloidal gel formation in polymer–colloid mixtures [53]. In the case of the RNP condensates, we expected a considerable alteration in the mesoscale dynamics, such as the biomolecular diffusion rate, with increasing PEG. Therefore, we next investigated the physical properties of the FUS^FL^ condensates as a function of PEG8000 concentration at a fixed protein concentration (10 μM; Figure 1b–d). We used two complementary methods to probe FUS^FL^ condensate material states: (a) Fluorescence recovery after photobleaching (FRAP), and (b) controlled fusion of suspended droplets using a dual-trap optical tweezer. Using FRAP, we measured the half-time of fluorescence recovery (τ12FRAP) of the fluorescently-tagged RNP (Alexa488-FUS) after photobleaching a circular region at the center of the droplet (Figure 1b,c and Figure S1). Analysis of the τ12FRAP and effective bleach radius (*r_e_*) allowed us to estimate an apparent diffusion coefficient (*D_app_*) of a fluorescently tagged RNP within the protein-dense phase (see SI methods and Figure S1). The fraction of the mobile phase was also estimated from FRAP data and was used as a relative measure of the viscoelastic properties of FUS^FL^ droplets [8]. With increasing PEG8000, our FRAP data revealed two distinct trends (Figure 1b–d): (i) An increase in the fluorescence recovery half-time and, hence, a concomitant decrease in molecular diffusivity dynamics; and (ii) a decrease in the fraction of the mobile phase. The observed molecular diffusion rate decreased nearly fourfold upon increasing the PEG8000 concentration from 0 to 150 mg/mL. Concurrently, total fluorescence recovery after bleaching decreased from 100% to ~40% for FUS^FL^ droplets. These data suggest that macromolecular crowding mediates a progressive transition of FUS^FL^ droplets from a viscous fluid state to a viscoelastic state, resulting in substantially arrested protein diffusion.

**Figure 2:**
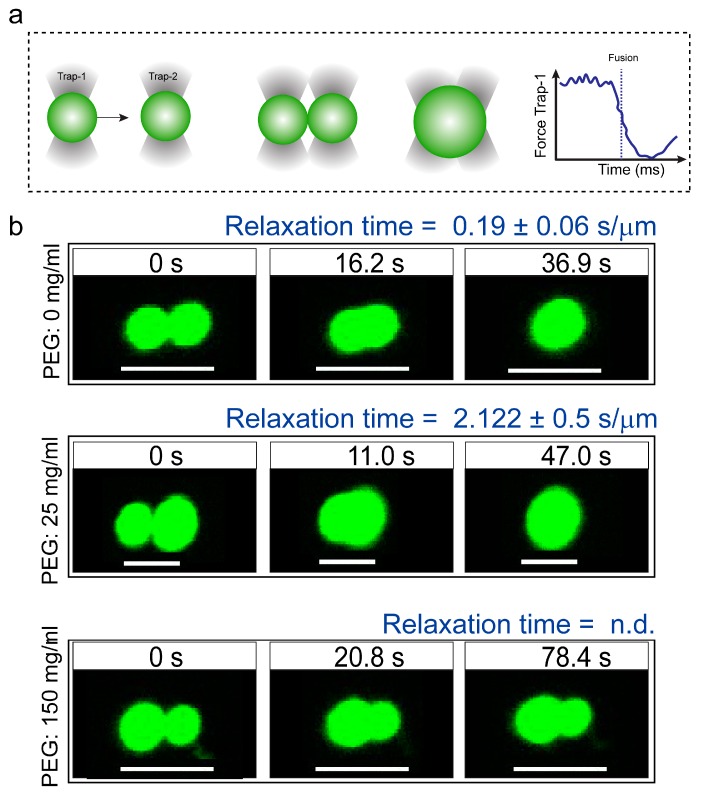
Coalescence dynamics of suspended condensates provide insight into FUS^FL^ droplet material states. (**a**) Experimental scheme of RNP condensate fusion using a dual-trap optical tweezer. (**b**) Controlled fusion of suspended FUS^FL^ droplets by the optical tweezer in the presence of 0 (top), 25 (middle), and 150 (bottom) mg/mL of PEG8000. The normalized relaxation times are indicated in each case. For 150 mg/mL PEG samples, droplets did not completely fuse. Scale bar = 5 μm. n.d.: not determined. Additionally, see Movies S1–3.

To gain further insight into the altered physical properties of FUS^FL^ droplets in the presence of macromolecular crowders, we performed quantitative droplet fusion experiments using a dual-trap optical tweezer. In these experiments, we used one laser beam to hold one RNP droplet at a fixed position while another RNP droplet, trapped by a second laser, was moved towards the first droplet at a constant velocity (Figure 2a and Figure S2) [47]. As these droplets were brought into proximity, liquid FUS^FL^ droplets coalesced rapidly in the absence of any crowders with a timescale of ~200 ms/μm (Figure 2, top panel; movie S1). In the presence of 150 mg/mL PEG8000, the fusion events of FUS^FL^ droplets were almost arrested (incomplete fusion in Figure 2, bottom panel; movie S3). Instead, we observed that FUS^FL^ droplets clustered at the optical trap under this condition (Figure S3). These observations are consistent with our FRAP results (Figure 1b–d). Taken together, our experimental data suggest that macromolecular crowding by PEG leads to a substantial hardening of FUS^FL^ droplets. We note that the observed alteration in the material properties of FUS^FL^ condensates occurred in the presence of crowding without any conclusive evidence for fiber formation (Figure S4).

### 2.2. The Effect of Macromolecular Crowding on RNP Condensation is Largely Independent of the LCD Sequence 

**Figure 3:**
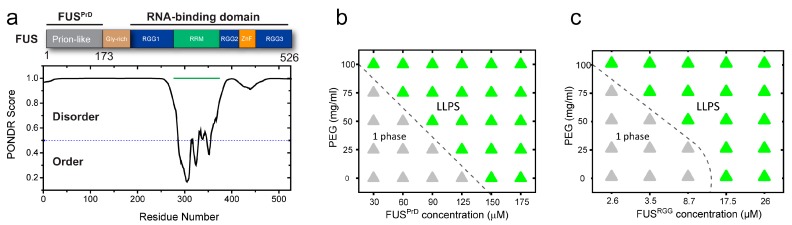
FUS^FL^ harbors distinct low-complexity disordered domains (LCDs) that individually undergo crowding-mediated liquid–liquid phase separation (LLPS). (**a**) Domain architecture of FUS; two distinct disordered domains are highlighted. The N-terminal FUS LCD has prion-like sequence features (FUS^PrD^) and C-terminal LCD is enriched in RGG-repeats (FUS^RGG^). The disorder prediction scores (using VSL2 algorithm [60]) are also shown in the bottom panel. (**b**,**c**) Effect of PEG on the LLPS of prion-like LCD and arginine-rich polycationic LCD (R-rich LCD). Shown here are the phase diagrams of FUS^PrD^ and FUS^RGG^ with PEG8000, respectively. The dotted lines indicate the liquid–liquid coexistence boundaries.

The phase separation of the FUS family of RNPs is assumed to be driven by enthalpy, that is, attractive protein–protein interactions, and, therefore, is sensitive to LCD sequence features that encode essential intermolecular interactions [7]. As such, recent studies focused on elucidating the sequence determinants of LLPS in RNPs, which revealed two major classes of LCDs [46,47]. These are (i) prion-like LCD (PrD), characterized by an overabundance in polar (S/G/Q) and aromatic (Y/F) residues but largely devoid of charged amino acids [1,2,7,8,41,54,55,56]; and (ii) arginine-rich polycationic LCD (R-rich LCD) [57], such as the disordered RGG-box sequences present in the RNA-binding domains of many RNPs [58]. In the ribonucleoprotein FUS, both of these LCD types (FUS^PrD^ and FUS^RGG^) are present as individual domains in the N- and C-termini of the protein, respectively (Figure 3a; Supplementary Table S1). To gain a mechanistic understanding of the impact of crowding on the physical properties of RNP droplets formed by these distinct LCDs, we next studied the phase behavior and physical properties of FUS^PrD^ and FUS^RGG^ condensates independently. FUS^PrD^ is intrinsically disordered, as predicted bioinformatically [59] (Figure 3a) and verified experimentally [49]. FUS^PrD^ is also known to form amyloids in vitro that contain dynamic β-sheet-rich structures [60,61], the formation of which is assumed to facilitate the droplet aging process [42]. While FUS^PrD^ has been implicated as the major driver of FUS LLPS [62], the role of FUS^RGG^ in RNP condensation is only beginning to be explored [47]. Similar disordered RGG-box regions have previously been shown to form homotypic condensates [63]. Here, to study the effect of crowding, we first constructed phase diagrams of PEG–LCD mixtures for these two distinct LCDs separately. Both LCD types underwent concentration-dependent reversible phase separation upon lowering the solution temperature (Figure S5), suggesting that there exists an upper critical solution temperature (UCST) for these two disordered domains, above which a homogeneous phase is energetically favored. The UCST phase behavior for the two LCDs also indicates that their phase separation is driven predominantly by favorable free energy change during self-association via attractive protein–protein interactions [7]. In presence of PEG8000, we observed that the phase separation of both FUS LCDs are facilitated. The isothermal phase diagram (at 22 ± 1 °C) analyses for FUS^PrD^ and FUS^RGG^ as a function of PEG8000 are presented in Figure 3. We observed that PEG decreased the protein condensation critical concentration by approximately fivefold for FUS^PrD^ in response to an increase in the PEG concentration from 0 to 100 mg/mL. A similar trend was also observed for FUS^RGG^, where the concentration required for the protein to undergo LLPS was decreased by approximately sevenfold in the presence of 100 mg/mL PEG. These results are consistent with the hypothesis that the crowder-mediated depletion force acts synergistically with the homotypic LCD–LCD interactions (see Note S1). 

**Figure 4:**
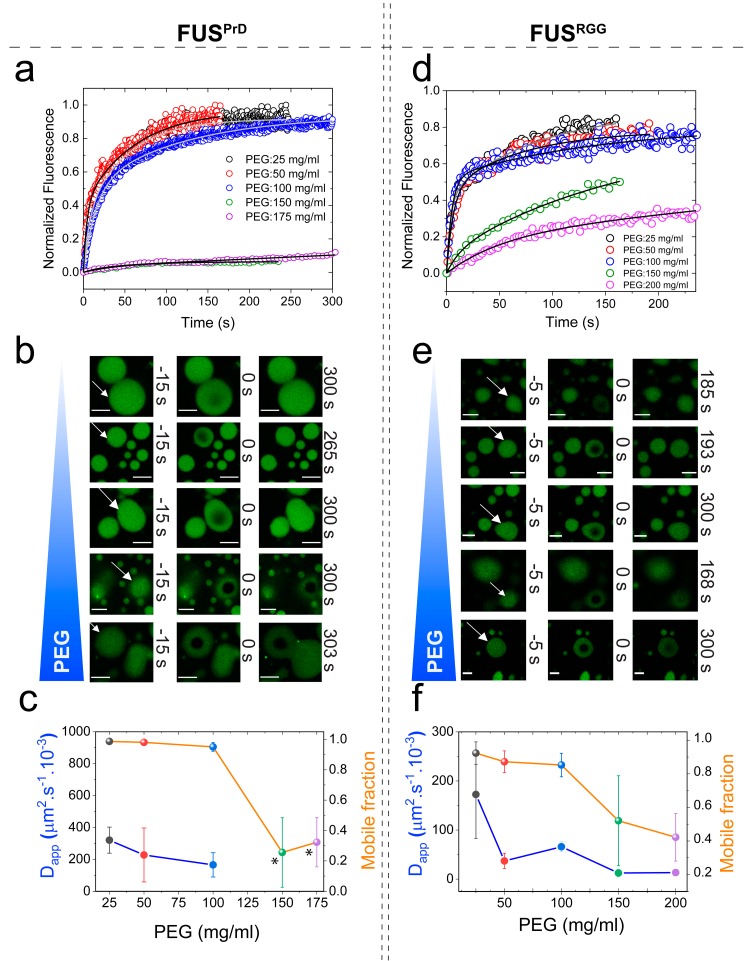
Macromolecular crowding tunes viscoelastic properties of both prion-like and R-rich LCDs. **(a**,**b**,**d**,**e**) Representative FRAP plots and images of FUS^PrD^/FUS^RGG^ droplets at variable concentrations of PEG8000, respectively. Scale bar = 8 μm in (**b**) and = 4 μm in (**e**). Negative time implies droplets in pre-bleaching state in (**b**,**e**). (**c**,**f**) Analyses of the FRAP data estimating apparent diffusion coefficients (D_app_) of respective LCDs within the condensed phase (left axis, blue) and the mobile phase fraction (right axis, orange) in respective cases. Due to a very low fraction of recovery, D_app_ estimation from the FRAP data for FUS^PrD^ droplets at 150 and 175 mg/mL PEG was omitted (indicated by the asterisks in (**c**)).

During the visualization of FUS^PrD^ and FUS^RGG^ condensates using a confocal fluorescence microscope, we noted that the well-dispersed LCD droplets underwent clustering at higher PEG concentrations (Figure S6). This observation indicates a crowding-dependent change of condensate physical properties. Therefore, we next investigated LCD droplet material properties in the presence of macromolecular crowders. Using Alexa488-labeled LCDs, we quantitatively analyzed fluorescence recovery after photobleaching in a well-defined region within FUS^PrD^/FUS^RGG^ droplets (Figure 4). The recovery of fluorescence was modelled based on the diffusion of LCDs within the respective condensed phases (Figure S1). For FUS^PrD^ droplets, FRAP analyses revealed that the diffusion rate decreased significantly with an increase in the PEG8000 from 0 to 175 mg/mL. Only ~20% fluorescence recovery was observed at ≥150 mg/mL PEG8000 in a timescale of 300 s after bleaching (Figure 4a–c). The FUS^RGG^ droplets showed a similar arrest in protein mobility at a high concentration of PEG8000 (Figure 4d–f), although the changes were observed to be more gradual compared to FUS^PrD^. We speculate that the differences observed in the FRAP trend between FUS^PrD^ and FUS^RGG^ as a function of crowder concentration is a manifestation of sequence-encoded distinct molecular interactions and polypeptide chain dynamics in respective LCDs. We also note that FUS^FL^ FRAP data (Figure 1b) showed a gradual change with increasing crowder concentration, similar to FUS^RGG^. Overall, these data suggest that both FUS^PrD^ and FUS^RGG^ droplets undergo progressive hardening with increasing PEG, despite their diverse sequence features and charge patterning.

### 2.3. Crowding Impact on the Material Properties of FUS Condensates Is Observed for a Broad Range of Crowders 

**Figure 5:**
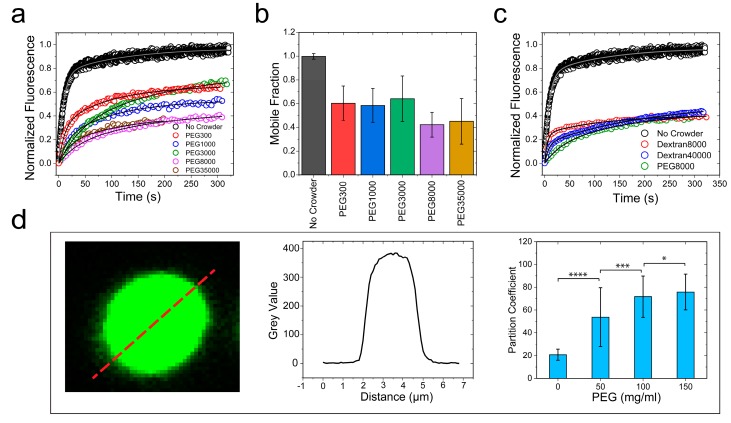
PEG and dextran produce similar effects on FUS^FL^ droplet physical properties. (**a**,**c**) Representative FRAP traces at 150 mg/mL PEG/dextran with a wide range of molecular weights, as indicated. The corresponding FRAP images and diffusion analyses are shown in Figure S7. (**b**) The fraction of the mobile phase decreased with increasing PEG molecular weight**.** (**d**) Increased ribonucleoprotein (RNP) partitioning within the FUS^FL^ droplets with increasing concentration of PEG8000, as probed by confocal image analysis. * *p*-value: 0.1–0.01, ** *p*-value: 0.01–0.001, *** *p*-value: 0.001–0.0001, **** *p*-value < 0.0001.

Macromolecular crowding in a cell arises from biopolymers with a plethora of sizes and shapes. In order to evaluate the excluded volume effect on RNP LLPS, it is necessary to use polymer crowders with variable chain lengths [25,64]. Therefore, we next considered the impact of crowders with different molecular weights on the FUS^FL^ condensation. To this end, we employed PEG polymers with molecular weights ranging from 300 to 35,000 gm/mol. FRAP experiments revealed that FUS^FL^ droplets were viscoelastic in the presence of all the crowders tested (crowder concentration = 150 mg/mL), with a significant degree of arrest in the dynamic exchange of the fluorescently tagged RNPs (Figure 5a and Figure S7b). Dextran, another widely utilized molecular crowder with a different chemical identity, also showed similar effects on FUS^FL^ condensates (Figure 5c and Figure S7d). These data collectively suggest that our observed viscoelastic tuning of FUS condensates is generally applicable to a broad range of polymer crowders and, therefore, represents a common effect of depletion interaction as induced by macromolecular crowding. 

## 3. Discussion

**Figure 6:**
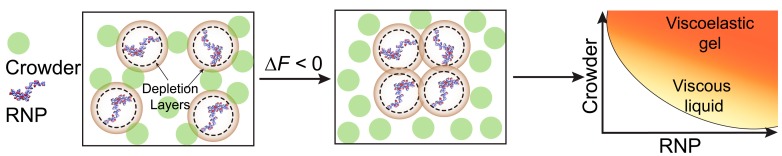
Schematic representation of depletion attraction: Proposed model showing crowder-mediated overlap of depletion layers as a driving force underlying RNP droplet formation and maturation into a gel-like phase in a crowded medium.

Intracellular RNP granules are phase-separated bodies that display characteristic dynamics of complex fluids [2,65]. The mesoscale physical properties of RNP condensates are important modulators of their functions [3]. Aberrant alterations of the droplet material states, such as age onset loss of granule fluidity and formation of solid-like assemblies, are implicated in various neurological disorders, including ALS and frontotemporal dementia (FTD) [42,46,50]. Over the past two years, considerable efforts have been dedicated to characterizing RNP sequence-encoded molecular interactions that control the material properties of the RNP condensates [47]. In this study, we considered the role of molecular crowding—a ubiquitous thermodynamic force in the cellular environment—on the RNP condensate dynamics. We experimentally showed that crowding, as mimicked by biocompatible “inert” polymers, not only lowered the LLPS boundary, but also substantially altered the exchange dynamics of the RNP within the condensed phase. We observed that the aforementioned effects of crowding on LCD-driven LLPS, namely facilitating liquid condensation and droplet hardening, were largely independent of respective disordered domain sequences. In other words, while the saturation concentration and the condensate physical properties are governed by polypeptide sequence composition and patterning, their alterations by molecular crowding were observed to be a common effect in both types of LCDs. To provide a mechanistic picture of the observed effect of crowding on the RNP condensation, we consider a thermodynamic model that describes the perturbation of protein–protein interactions by a crowder in light of the well-established excluded volume effect [30,33]. According to this model, the introduction of a polymer crowder in the RNP–buffer solution leads to an isotropic inter-protein attraction by the depletion force (see Note S1), which acts in tandem with the intrinsic LCD–LCD homotypic attraction. The physical origin of the depletion attraction is the exclusion of the center of mass of a crowder molecule from a region surrounding an RNP molecule, which is typically called the depletion layer (Figure 6). The depletion layer thickness is directly proportional to the hydrodynamic radius of the polymer crowder [34]. This simple thermodynamic model predicts that increasing crowder concentration in the solution will increase the overlap of the RNP depletion layers in order to produce excess free volume available to the polymer crowders (Figure 6). This implies that the net attractive LCD–LCD attraction is enhanced by macromolecular crowding, which lowers the critical concentration of RNP LLPS and results in hardening of RNP condensates. Mathematically speaking, as the concentration of PEG increases, the chemical-potential derivative, ${\left(\frac{\partial \mu_{protein}}{\partial c_{protein}}\right)}_{T}$, which provides a thermodynamic basis of inter-protein attraction, decreases monotonically (see Equation (4) in Note S1). This quantity defines the spinodal curve, which is the boundary between the stable and unstable regions of the protein–crowder–buffer system (Figure S8). When ${\left(\frac{\partial \mu_{protein}}{\partial c_{protein}}\right)}_{T,c_{pol}}>0$, the system is stable as a homogeneous solution, whereas it phase-separates into two coexisting liquids if ${\left(\frac{\partial \mu_{protein}}{\partial c_{pro}tein}\right)}_{T,c_{pol}}<0$. Therefore, the net effect of the crowder may be physically interpreted as an effective increase in FUS–FUS attraction, manifested by depletion interactions. One key prediction of this model is that RNP partitioning in the dense phase should increase with increasing crowding (Figure S8). An increase in partitioning theoretically means a longer tie line that causes the volume fraction of the RNP in the dense phase to increase with a simultaneous decrease in the RNP volume fraction in the dilute phase. This is directly related to the strength of the inter-protein interaction parameter which increases the curvature of the free energy surface, pushing the coexisting states further apart (Figure S8). This was indeed experimentally verified for FUS^FL^ condensates using confocal fluorescence image analysis, which indicated that the RNP partition increased by about fourfold in response to an increase in the PEG8000 concentration from 0 to 150 mg/mL (Figure 5d). This observed increase in partition coefficient suggests that FUS^FL^ droplets become more dense with increasing crowder concentration. Such an increase in partitioning may also influence the rate at which FUS^FL^ condensates mature into a solid phase, which has been previously shown to accelerate with increasing FUS^FL^ concentration [42].

In summary, we demonstrated that depletion interaction, as induced by macromolecular crowding, continuously tunes the physical properties of RNP condensates ranging from purely viscous fluids to viscoelastic gel-like states. Alteration in the material properties of phase-separated membraneless compartments inside cells has been previously observed both in normal physiology and pathology [42,66,67]. Our results suggest that the hardening of RNP condensate is considerably influenced by the entropic forces in the crowded cellular environment. Several recent reports focused on identifying key RNP sequence features, post-translational protein modifications, and the role of RNA/protein partner binding that contribute to the gelation of RNP droplets [11,46,47]. Based on the data presented here, we postulate that generalized thermodynamic forces that can tune effective RNP homotypic interactions are likely to influence the rate at which physiological condensates mature into a viscoelastic gel.

## 4. Materials and Methods

### 4.1. Protein Samples

Codon optimized wild-type full-length FUS (FUS^FL^), prion-like domain of FUS (FUS^PrD^; AA: 1-173), and the RNA-binding domain of FUS without the zinc-finger domain (FUS^RGG^:211-526Δ422-453) were gene synthesized by GenScript USA Inc. (Piscataway, NJ, USA) and cloned into (cloning site: SspI-BamHI) pET His6 MBP N10 TEV LIC cloning vector (2C-T). The plasmid vector was a gift from Scott Gradia (Addgene plasmid # 29706). TEV-cleaved proteins contained three exogeneous amino acids (SNI) at its N-termini. *E. coli* cells (BL21(DE3)) were transformed with the plasmids containing FUS^FL^ and its variants in respective cases. Transformed cells were induced with IPTG (0.5 mM final concentration) at OD = 0.6–0.8 and further grown for an additional 3–5 h at 30 °C. Protein extraction was performed using a french press in lysis buffer (50 mM Tris-HCl, 10 mM imidazole, 1 M KCl, pH 8.0) containing protease inhibitor cocktail (Roche). Cell debris were removed by centrifugation. His-tag proteins were purified from the crude cell lysate using Ni-NTA agarose matrix (Qiagen Inc, Valencia, CA, USA) by gravity-flow chromatography following the manufacturer’s protocol with the following modifications: the wash buffer included 1.5 M KCl to disrupt nucleic acid binding to the recombinant protein [68,69], which was eluted with elution buffer containing 250 mM imidazole and 150 mM NaCl. The purity of the eluted protein samples was checked using A_280_/A_260_ measurements (to rule out presence of nucleic acids), and by polyacrylamide gel electrophoresis (PAGE) and Coomassie blue staining. The eluates (individual or pooled) were dialyzed against 25 mM Tris-HCl, pH 7.5 buffer containing 10% glycerol. The concentration of the protein samples were determined by absorbance at 280 nm using the following extinction coefficients: 103,600 M^−1^.cm^−1^ for FUS^PrD^-MBP, 86,750 M^−1^·cm^−1^ for FUS^RGG^-MBP, and 138,000 M^−1^·cm^−1^ for FUS^FL^-MBP (https://web.expasy.org/protparam). The protein samples were flash frozen in small aliquotes and stored in −80 °C. 

### 4.2. Fluorescence Labeling

The S86C variant of FUS^PrD^ and A313C variant of the FUS^RGG^ were expressed and purified using an identical protocol as described above for the WT protein, except one modification: all buffers contained 2 mM DTT. The protein samples were fluorescently labeled with Alexa488 dye (C5-maleimide derivative, Molecular Probes) using Cys-maleimide chemistry as described in our earlier work [23]. The labeling efficiency for all samples were observed to be ≥ 90% (UV-Vis absorption measurements), and no additional attempt was made to purifiy them further, given that only labeled protein is observed in the fluorescence experiments.

### 4.3. Sample Preparation for Phase Separation Measurements

All of the protein samples were buffer exchanged into the phase separation buffer (25 mM Tris-HCl, pH 7.5) containing 150 mM NaCl unless otherwise noted. Prior to performing phase separation measurements, the His_6_-MBP-N10 tag was removed by the action of TEV protease (1:25 ratio) (GenScript USA Inc.) for 1 h at 30 °C. The completion of the cleavage reaction was judged by polyacrylamide gel electrophoresis (PAGE) and Coomassie blue staining. 

### 4.4. Phase Diagram Analysis

Phase diagrams were constructed by turbidity measurements at 350 nm using a NanoDrop oneC UV-Vis spectrophotometer at room temperature (22 ± 1 °C). Desired amounts of PEG solutions were added to the protein solutions from a 35% (*w*/*v*) stock in nuclease-free water with appropriate salt concentrations. Each sample was incubated ~120 s prior to turbidity measurements using a 1 mm optical path length. Simultaneously, visualization of protein droplets (or lack thereof) was performed using a Primo-vert inverted iLED microscope (Zeiss), equipped with a Zeiss Axiocam 503 monochrome camera. A global analysis of the turbidity and microscopy data was performed to construct phase diagrams in respective cases based on a simple binary criterion that identifies if droplets were present at a given protein/PEG concentration.

### 4.5. Confocal Fluorescence Microscopy

The fluorescence and DIC imaging were performed using a Zeiss LSM 710 laser scanning confocal microscope, equipped with a 63× oil immersion objective (Plan-Apochromat 63×/1.4 oil DIC M27) and a Zeiss Primovert inverted microscope. Samples were prepared and imaged using tween-coated (20% *v*/*v*) Nunc Lab-Tek Chambered Coverglass (ThermoFisher Scientific Inc.) at room temperature (22 ± 1 °C) unless otherwise noted, with ~ 1% labeled protein samples within the mixture of unlabeled proteins. All the samples were allowed to equilibrate in the chambered coverglass for ~30–45 min before imaging. For Alexa488-labeled samples, the excitation and emission wavelengths were 488 nm/503–549 nm; for Alexa594-labeled samples, the excitation and emission wavelengths were 595 nm/602–632 nm. Fluorescence recovery after photobleaching (FRAP) experiments were performed using the same confocal set up. The images and data were analyzed using Fiji software [70] and the FRAP curves were plotted and analyzed using origin software (OriginPro 2018).

### 4.6. Fluorescence Recovery after Photobleaching

FRAP experiments were performed using Zeiss LSM 710 laser scanning confocal microscope as described above. A circular region of interest (ROI) was bleached with 2–5 iterations of scanning using 100% laser power for a total time of 2–18 s. Fluorescence intensity changes with time were recorded for three different ROIs (bleached droplet, reference droplet, and background) for approximately 300 s or until the bleached ROI recovered and reached an equilibrium state. Data analyses were performed using Fiji software and MATLAB.

The fluorescence intensities from bleached ROI were corrected for photofading by multiplying with a correction factor obtained from reference ROI as follows:

$$C_{f}=\frac{R_{i}}{R\left(t\right)}$$

$$I_{corrected}=C_{f}\times I_{bleached}\left(t\right)$$

$R_{i}$: Initial intensity of reference droplet; R(t): Intensity of reference ROI at time *t*; $C_{f}$: Correction factor; $I_{bleached}\left(t\right)$: Intensity of bleached ROI at time *t*; $I_{corrected}\left(t\right)$: Intensity of bleached ROI (corrected for photofading) at time *t*.

The corrected itensities were shifted to set the immediate post-bleach point to zero.

$$I_{shifted}\left(t\right)=I_{corrected}-\mathrm{min.}\;\mathrm{value}\;\mathrm{of}\;I\left(t\right)$$

which were then normalized.

$$I_{Normalized}\left(t\right)=\frac{I_{shifted}\left(t\right)}{\mathrm{max.}\;\mathrm{value}\;\mathrm{of}\;I_{shifted}\left(t\right)}$$

This normalized intensity post-bleach was plotted vs. time and fitted with a single exponential y=A(1−exp(−t/τ) using MATLAB. Half time of recovery ($\tau_{1/2})$ was obtained from the fitting parameter. To improve the goodness of the fit, two-exponential fit $y=A(1-\exp(-t/\tau_{A})+B(1-\exp(-t/\tau_{B})$ was also used [71]. Half time of recovery ($\tau_{1/2})$ was obtained graphically for the latter (Figure S1c,d). 

To account for diffusion during bleaching, instead of simply using user defined bleach radius (*r_n_*) for diffusion coefficient calculations, an effective radius (*r_e_*) from the immediate post bleach frame was calculated by taking a profile across bleached ROI in Fiji. The normalized fluorescence intensities were plotted with distance and fitted with an exponential of a Gaussian laser profile using MATLAB, as previously described [72,73].

(1) $$f\left(x\right)=\exp(-Kexp\left(\frac{-2{\left(x-b\right)}^{2}}{r_{e}{}^{2}}\right)$$

*r_e_* obtained from the fitting is the effective radius which corresponds to half width at 86 % of bleach depth K (Figure S1a,b).

The apparent diffusion coefficient [72] was calculated using the following equation:

(2) $$D=\frac{r_{e}{}^{2}+r_{n}{}^{2}}{8\tau_{1/2}}$$

The mobile fraction [72,73] was calculated using the following formula:

(3) $$M_{f}=\frac{I_{\infty }-I_{0}}{I_{i}-I_{0}}$$

$I_{\infty }$: Fluorescence intensity after recovery; $I_{0}$: Fluorescence intensity immediately after bleach; $I_{i}$: Fluorescence intensity before the bleach.

### 4.7. Coalescence of Suspended Droplets by Dual-Trap Optical Tweezer

Controlled fusion assays were conducted to investigate changes in the material properties of the FUS^FL^ condensates as a function of crowder concentration. The samples were injected into a 25 mm × 75 mm × 0.1 mm single chamber custom-made flow cell. Samples at 10 μM protein concentration were prepared with different PEG8000 concentrations and equlibrated for ~30 min at room temperature. The trap-induced fusion was done using a dual-trap optical tweezer system coupled with laser scanning confocal fluorescence microscope (LUMICKS^TM^ C-trap). In a typical fusion experiment, two droplets were trapped with a 1064 nm laser with minimum power to reduce heating effect. The trapping of droplets was acheived due to a difference in the refractive index between the condensate and the dilute phase. After trapping, one droplet is brought into contact with the other droplet at a constant velocity of 40 nm/s. The trap remains traveling at that velocity until the fusion is completed and the final droplet relaxes to a spherical shape. The force on the moving trap was recorded at 78.4 kHz sampling frequency and analyzed using a fusion relaxation model [45]. The following equation was used to fit the force-time curve:

(4) $$F=ae^{\left(-t/\tau \right)}+bt+c$$

where the parameter τ is the fusion relaxation time. We scaled the fusion time by the average of the radii of the two droplets for every event. The linear term in the model is added to account for the constant trap velocity. First, we did a control experiment on a slow fusion sample and the relaxation times were obtained both from force curves as well as from aspect ratio analysis using fluorescence images [74]. The results were in good quantitative agreement (data not shown). For FUS^FL^ samples, at least 15 droplet fusion events were collected for each PEG concentration and the scaled relaxation times were averaged. An example of a typical normalized force curve with the fitted model is shown in Figure S2.

### 4.8. Partition Analysis

Phase separated samples containing appropriate amount of fluorescently tagged protein were placed in a single-chambered custom-made flow cell (see Section 4.7). Droplets were imaged at the surface using laser scanning confocal fluorescence microscope using 60× water-immersion objective (LUMICKS^TM^, C-trap). Images were analyzed using Fiji software. To calculate the partition coefficient, the mean intensity of the entire droplet was divided by mean background intensity for several droplets per sample for statistical accuracy using Excel. Statistical analysis were carried out using MATLAB.

### 4.9. Thioflavin T Assay

To probe for amyloid-like structure formation within FUS^FL^ condensates in presence of PEG8000 (150 mg/mL), we used a well-known amyloid reporter dye-Thioflavin T (ThT) [14]. 2–10 μM ThT probe was premixed with the experimental buffer (25 mM Tris. HCl, pH 7.5, 150 mM NaCl) used for FUS^FL^ (10 μM) droplet formation in presence of the crowder. Simultaneous fluorescence imaging of FUS droplets were performed by using Alex594 labeled FUS^PrD^. Fluorescence imaging was performed using a Zeiss LSM 710 laser scanning confocal microscope. The excitation and emission wavelengths were 458 nm and 490 nm, respectively, with the exictation laser set at the same power as the Alexa594 channel.

## Supplementary Materials

The following are available online at https://www.mdpi.com/2218-273X/9/2/71/s1. Table S1: Amino acid sequences of various constructs. Figure S1: Determination of biomolecular diffusion by fluorescence recovery after photobleaching (FRAP), Figure S2: Representative normalized force relaxation curve during trap-induced coalescence of suspended FUS^FL^ droplets, Figure S3: Aggregation of FUS^FL^ condensates in the optical trap in presence of 150 mg/mL PEG8000, Figure S4: FUS^FL^ condensates are ThT negative, Figure S5: FUS^LCD^ condensation is reversible and exhibit upper critical solution temperature (UCST), Figure S6: Clustering and morphological changes of FUS^PrD^ droplets with increasing concentration of PEG8000, Figure S7: FRAP analyses of FUS droplets in presence of various crowders, Figure S8: Representation of relative free-energy curves as a function of increasing crowder concentration showing transition of a stable homogeneous solution of FUS into a phase separated state, Note S1: A thermodynamic model describing the effect of a polymer crowder on the LLPS of FUS, Movie S1: Controlled fusion of suspended FUS^FL^ droplets by a dual-trap optical tweezer in the presence of 0 mg/mL of PEG8000, Movie S2: Controlled fusion of suspended FUS^FL^ droplets by a dual-trap optical tweezer in the presence of 25 mg/mL of PEG8000, Movie S3: Controlled fusion of suspended FUS^FL^ droplets by a dual-trap optical tweezer in the presence of 150 mg/mL of PEG8000.
