# Supplementary material for: Molecular Crowding Tunes Material States of Ribonucleoprotein Condensates
Source: Biomolecules. 2019 Feb 19;9(2):71. doi: 10.3390/biom9020071 (PMC6406554; doi:10.3390/biom9020071)
Supplement: Supplementary file 1 [file biomolecules-09-00071-s001.zip › Supplementary Information_final.docx]

**Supplementary Information**

Molecular Crowding Tunes Material States of Ribonucleoprotein Condensates

**Taranpreet Kaur^1^, Ibraheem Alshareedah^1^, Wei Wang^1^, Jason Ngo^1^, Mahdi Muhammad Moosa^2^, Priya R. Banerjee^1 *^**

^1^ Department of Physics, University at Buffalo, SUNY, NY, 14260, USA

^2^ Department of Pharmacology and Chemical Biology, Baylor College of Medicine, Houston, TX 77030, USA

* Corresponding author: Priya R. Banerjee (prbanerj@buffalo.edu)

**Supplementary Table-1**

| Construct Name | Amino acid sequence |
| --- | --- |
| FUS^PrD^ | MASNDYTQQATQSYGAYPTQPGQGYSQQSSQPYGQQSYSGYSQSTDTSGYGQSSYSSYGQSQNSYGTQSTPQGYGSTGGYGSSQSSQSSYGQQSSYPGYGQQPAPSSTSGSYGSSSQSSSYGQPQSGSYSQQPSYGGQQQSYGQQQSYNPPQGYGQQNQYNSSSGGGGGG GGG |
| FUS^RGG^ | QDRGGRGRGGSGGGGGGGGGGYNRSSGGYEPRGRGGGRGGRGGMGGSDRGGFNKFGGPRDQGSRHDSEQDNSDNNTIFVQGLGENVTIESVADYFKQIGIIKTNKKTGQPMINLYTDRETGKLKGEATVSFDDPPSAKAAIDWFDGKEFSGNPIKVSFATRRADFNRGGGNGRGGRGRGGPMGRGGYGGGGSGGGGRGGFPSGGGGGGGQQGPGGGPGGSHMGGNYGDDRRGGRGGYDRGGYRGRGGDRGGFRGGRGGGDRGGFGPGKMDSRGEHRQDRRERPY |

**Supplementary Figures**





Figure S1: Determination of biomolecular diffusion by fluorescence recovery after photobleaching (FRAP). a) Immediate post bleach frame with user defined ROI (yellow circle; rn = nominal radius). b) Normalized intensity profile across the bleached ROI to estimate the effective radius of bleaching (re). Black points are the data; red line is a fit to an exponential of a Gaussian laser profile using equation-1. c) Normalized FRAP curve (black points) fitted with a single exponential funcion (red line). d) Normalized FRAP curve (black points) fitted with a double exponential function (red line). The residuals are shown in respective cases, justifying our choice of a double exponential function to determine the $\tau_{1/2}$.


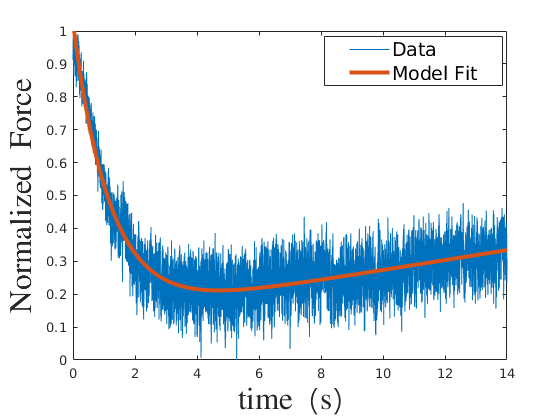


Figure S2: Representative normalized force relaxation curve during trap-induced coalescence of suspended FUSFL droplets. The data (blue trace) is fitted (red line) using a model described by equation-4.


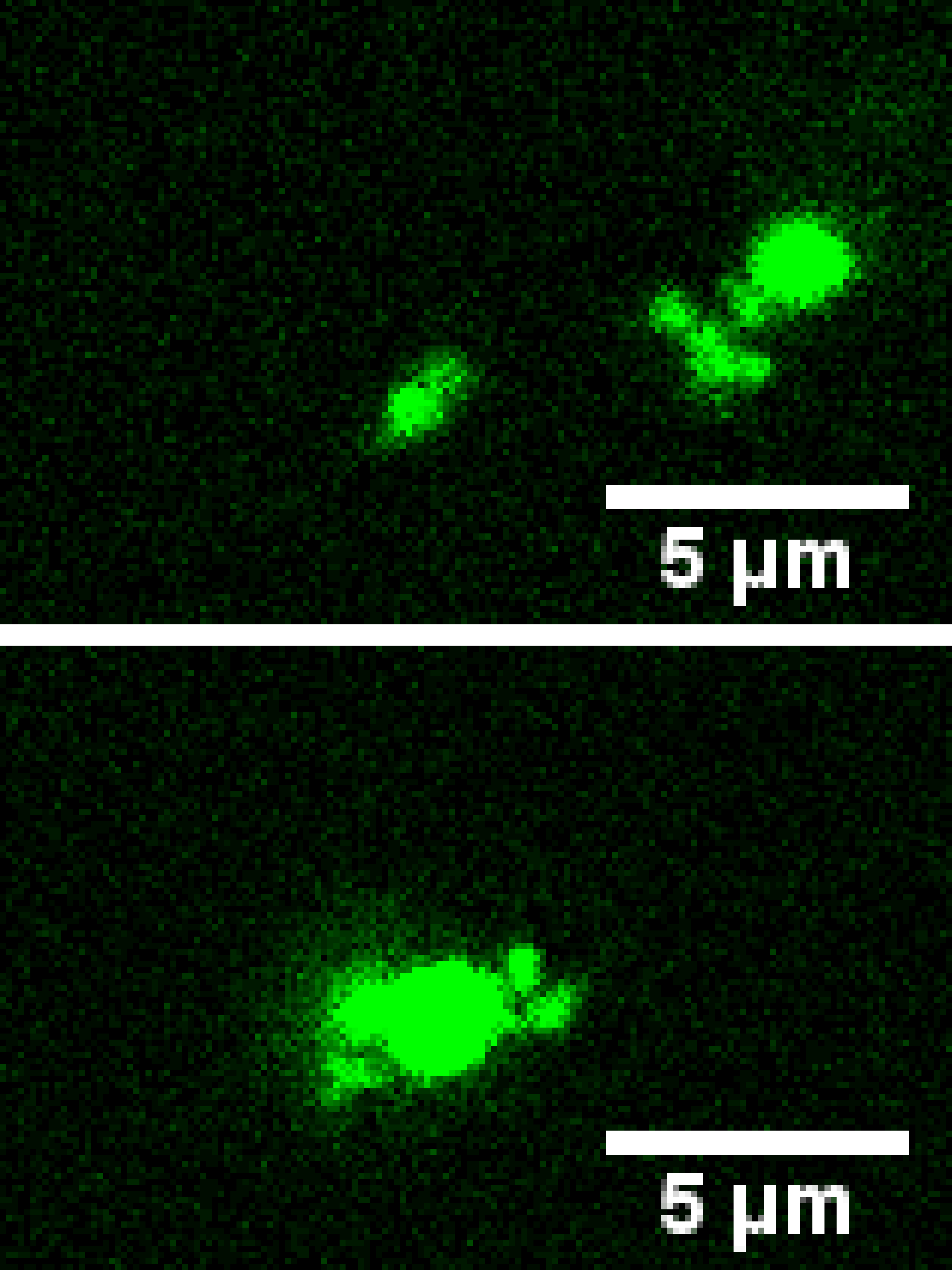


Figure S3: Aggregation of FUSFL condensates in the optical trap in presence of 150 mg/ml PEG8000. Two images (top and bottom) correspond to two independent measurements. The green color corresponds to Alexa488-labeled FUSPrD. FUS^FL^ concentration = 10 µM.


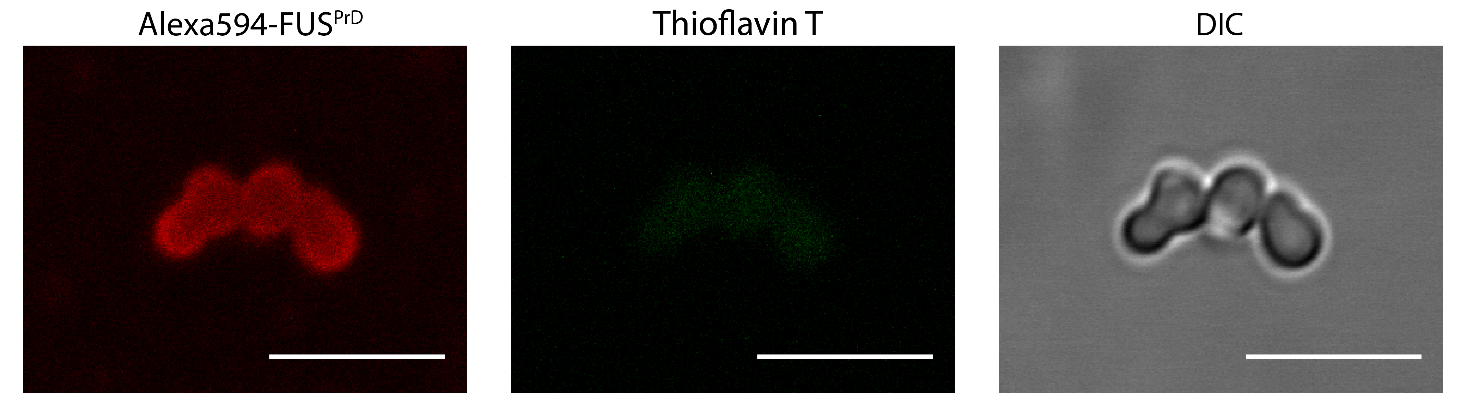


Figure S4: FUSFL condensates are ThT negative. The red color corresponds to Alexa594-labeled FUSPrD. FUS^FL^ concentration = 10 µM with 150 mg/ml PEG. Scale bar = 5µm


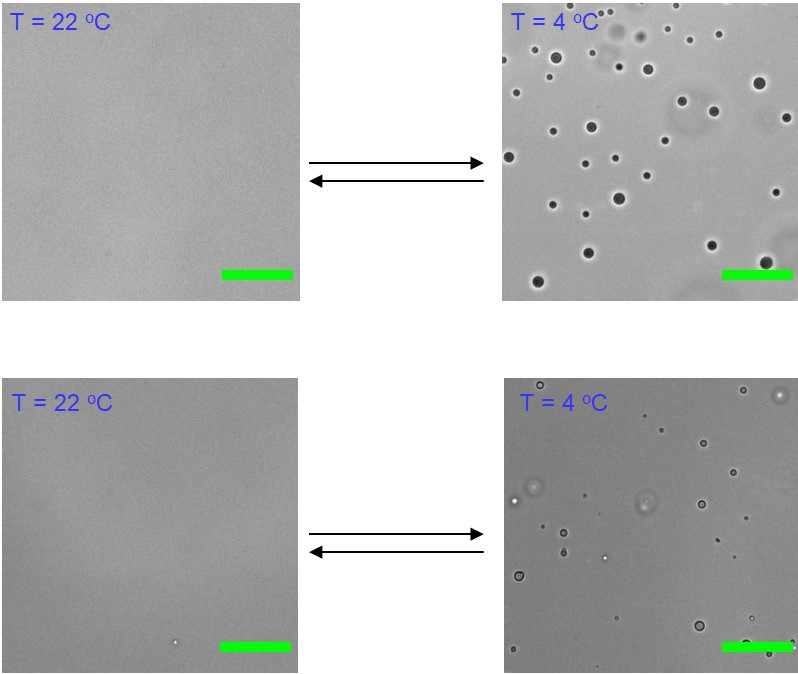


Figure S5: FUSLCD condensation is reversible and exhibit upper critical solution temperature (UCST). Effect of temperature on FUSPrD (100 µM; top two panels) and FUSRGG (25 µM; bottom two panels) LLPS, revealing UCST phase behavior. The bright-field images were collected using protein samples in 25 mM Tris.HCl buffer, pH 7.5, 150 mM NaCl. Scale bar = 25 µm.


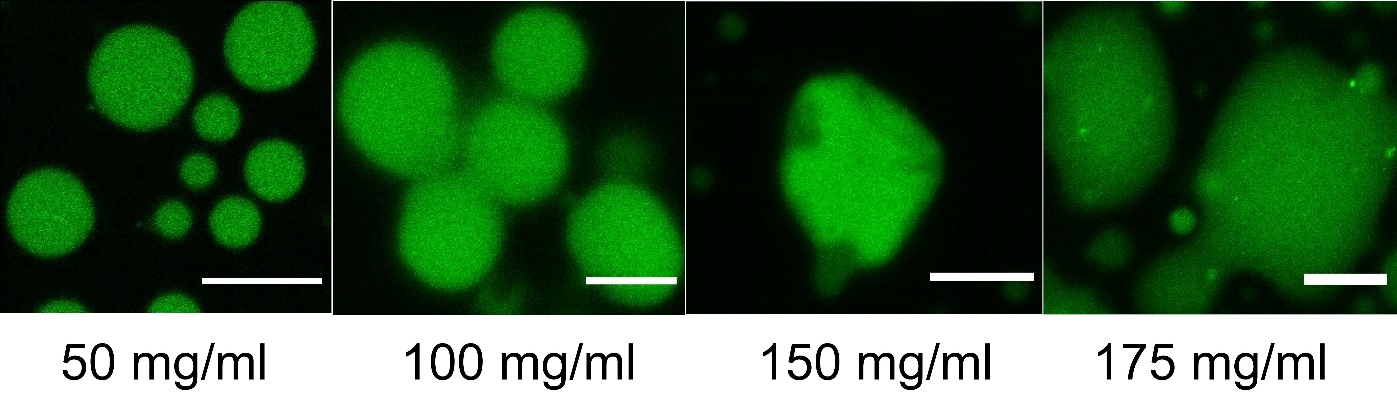


Figure S6: Clustering and morphological changes of FUSPrD droplets with increasing concentration of PEG8000. PEG concentrations are indicated in the figure. Protein concentration = 335 µM. Scale bar

=10 µm.


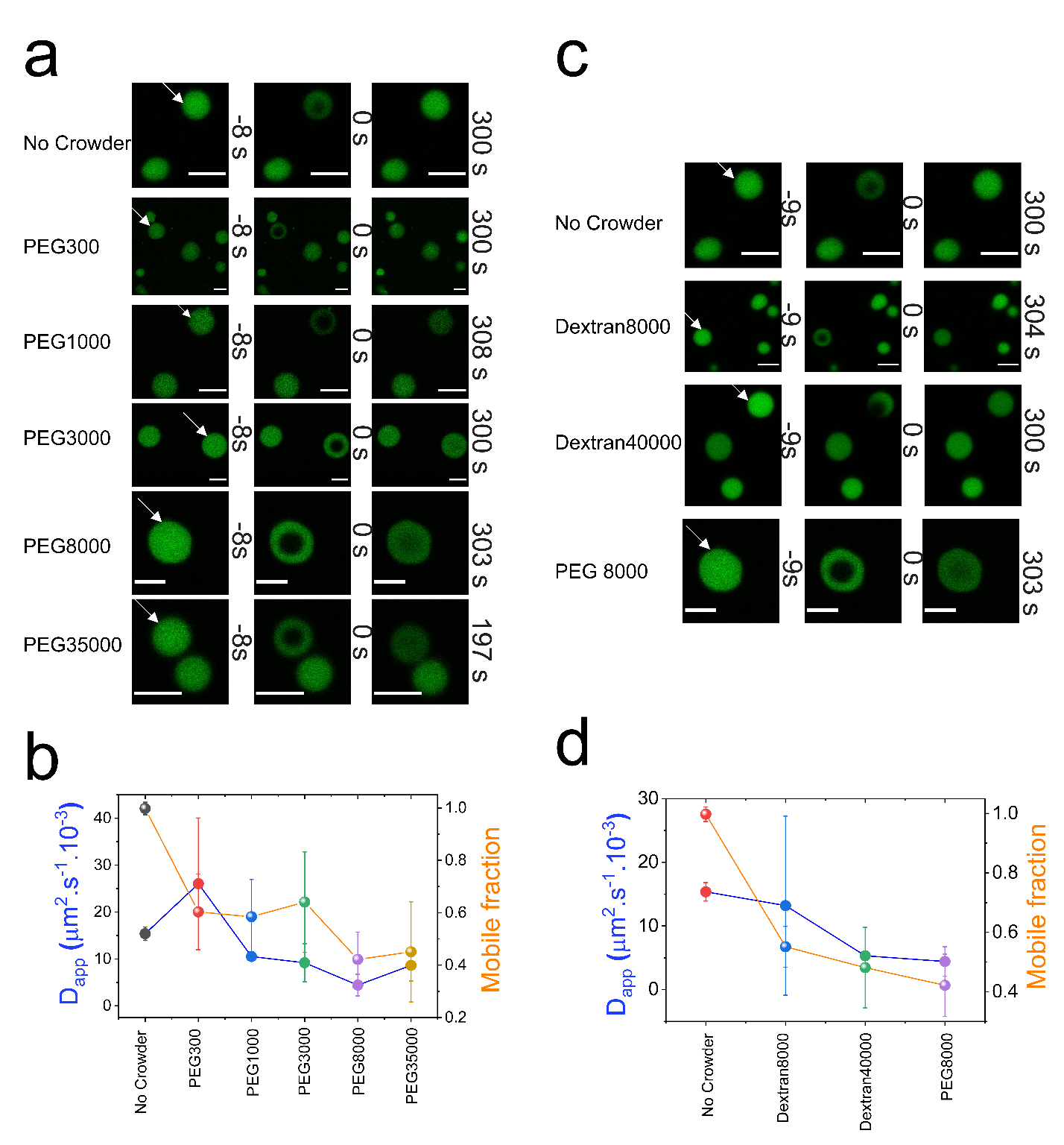


Figure S7: FRAP analysis of FUS^FL^ droplets in presence of various crowders. a) & c). Representative FRAP images of FUS droplets in presence of PEG and dextran as polymer crowders with different molecular weights, respectively. Crowder concentration used in each case: 150 mg/ml. Scale bar=4 µm. b) & d). Corresponding analysis of the FRAP results to quantify molecular diffusion (blue; right axis) and the fraction of mobile phase (orange; left axis).


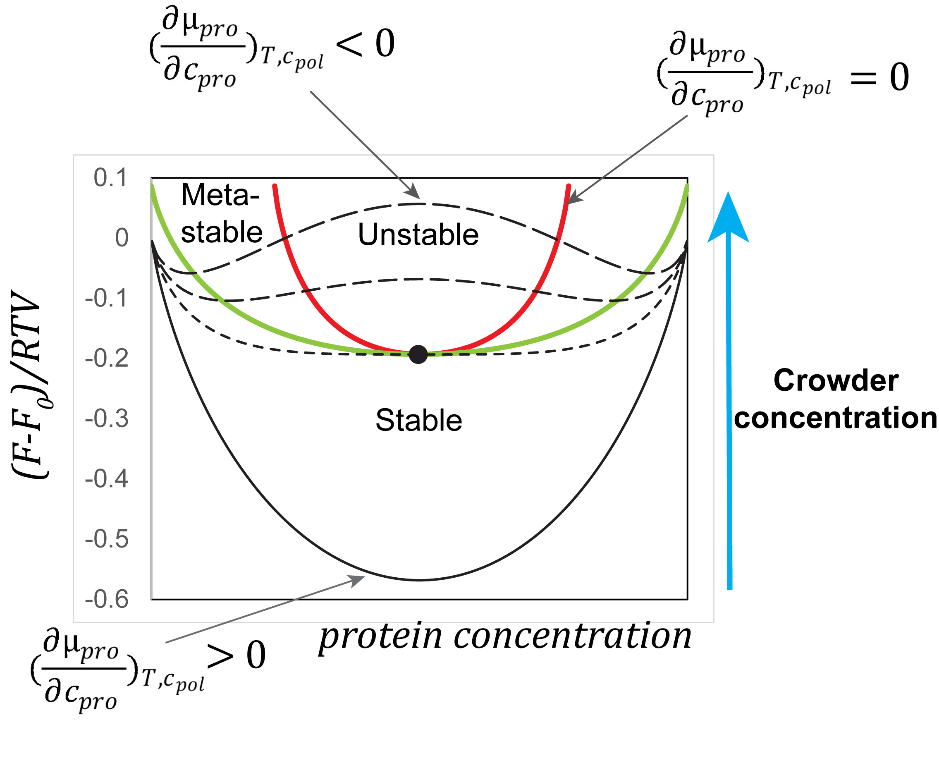


Figure S8: Simulated relative free-energy curves as a function of increasing crowder concentration showing transition of a stable homogeneous solution of FUS^FL^ into a phase separated state. Shown here are four free energy plots (black-lines). The red line shows the locus of the spinodal boundary, which separates unstable regions from metastable regions of the mixture. The green line is the coexistence boundary, which specifies the composition of the dense phase and the dilute phase. With increasing crowding, this model predicts that the protein partitioning in the dense phase should increase. This is experimentally observed for FUS^FL^ condensates and shown in Fig. 5c (*main-text*).

SI Note-1

*A thermodynamic model describing the effect of a polymer crowder on the LLPS of FUS*

To describe the effect of a polymer crowder on the LLPS of FUS-buffer system and to analyze the impact of crowder concentration on the effective FUS-FUS interactions, we consider a thermodynamic perturbation theory that was previously developed for gammaD crystallin-buffer-PEG mixture [1,2]. Using the subscripts “pro” to denote the protein and “pol” to denote the polymer crowder, we define the reduced free energy of the system: *f* = $(F-F_{0})/RTV$; F is the Helmholtz free energy of FUS-crowder-buffer system; F_0_ is called the standard free energy and is given by: F_0_ = $c_{pro}.\mu_{pro}^{0}+ c_{pol}.\mu_{pol}^{0}$; $\mu_{pro}^{0}$ and $\mu_{pol}^{0}$ are standard chemical potentials, R = ideal gas constant.

The first order approximation of the free energy with respect to the polymer crowder concentration can be expressed as [2]:

$f\left( c_{pro}, c_{pol}, T \right)= f\left( c_{pro}, c_{pol}=0, T \right)+ c_{pol}.ln\frac{c_{pol}}{e}+ c_{pol}{(\frac{{\partial f}^{ex}}{\partial c_{pol}})}_{c_{pro}, c_{pol}=0, T}+\ldots$ (1)

Here the 1^st^ term on the right hand side, $f\left( c_{pro}, c_{pol}=0, T \right)$, is the reduced free energy of the FUS-buffer binary mixture in absence of any crowder. The 2nd term signifies a change in the mixing entropy of the system due to the addition of PEG, whereas the 3^rd^ quantity accounts for the changes in the reduced free energy as a function of crowder concentration. For simplicity, we don’t consider the contribution of higher order terms, which is the case for ideal polymer crowders. This form of equation-1 can be used to study the variance of protein-protein attraction by a polymer crowder in light of the well-established excluded volume model [3]. The model is based on the consideration that the center of mass of a crowder molecule is excluded from a region surrounding a protein molecule, which is called depletion layer, due to pure steric forces. The depletion layer thickness is directly proportional to the radius of gyration (R_g_) of the polymer crowder. In such a case [2,4], a free volume fraction parameter ($\alpha)$ can be defined that signifies the fraction of volume available to the polymer crowders, $\alpha= exp(-{(\frac{{\partial f}^{ex}}{\partial c_{pol}})}_{c_{pro},c_{pol}=0, T})$. This definition helps us to re-write equation-1 in the following form:

$f\left( c_{pro}, c_{pol}, T \right)= f\left( c_{pro}, c_{pol}=0, T \right)+ c_{pol}.ln\frac{c_{pol}}{e}- c_{pol}.ln\alpha$ (2)

The reduced protein chemical potential of FUS-crowder-buffer system is given by:

$\mu_{pro}(c_{pro}, c_{pol}, T)=\left( \frac{\partial f}{\partial c_{pro}} \right)_{c_{pol}, T}= \mu_{pro}(cpro,cpol=0,T)- \frac{c_{pol}}{\alpha}{(\frac{\partial\alpha}{\partial c_{pro}})}_{T}$ (3)

The quantity, $\mu_{pro}(cpro,cpol=0,T)$ is the reduced protein chemical potential of FUS-buffer system in absence of any crowders. Differentiation of equation-3 with respect to $c_{pro}$ allows us to derive an expression for effective protein-protein interactions in presence of a crowder:

${(\frac{\partial\mu_{pro}}{\partial c_{pro}})}_{T,c_{pol}}= {(\frac{\partial\mu_{pro}(pro,cpol=0,T)}{\partial c_{pro}})}_{T}- \frac{c_{pol}}{\alpha}\left( \frac{\partial^{2}\alpha}{\partial{c_{pro}}^{2}} \right)_{T}=\mu_{binary}^{'}- \frac{c_{pol}}{\alpha}\left( \frac{\partial^{2}\alpha}{\partial{c_{pro}}^{2}} \right)_{T}$ (4)

The quantities, ${(\frac{\partial\mu_{pro}}{\partial c_{pro}})}_{T,c_{pol}}$ and $\mu_{binary}^{'}$ are related to the FUS-FUS interactions, in presence and absence of a crowder. They also mathematically define the spinodal curve, which is the boundary between the stable and unstable regions of the protein-crowder-buffer system (Fig. S8). When, ${(\frac{\partial\mu_{pro}}{\partial c_{pro}})}_{T,c_{pol}}>0$, the system is stable as a homogeneous solution, whereas it phase separates into two coexisting liquids if ${(\frac{\partial\mu_{pro}}{\partial c_{pro}})}_{T,c_{pol}}<0$. The critical point is defined as ${(\frac{\partial\mu_{pro}}{\partial c_{pro}})}_{T,c_{pol}}$= 0. In equation-4; the quantity, ${(\frac{\partial^{2}\alpha}{\partial{c_{pro}}^{2}})}_{T}$, describes the change in free volume fraction due to overlap of the adjacent depletion layers. Since free volume available to crowder molecules increases with increasing overlap of the depletion layers, ${(\frac{\partial^{2}\alpha}{\partial{c_{pro}}^{2}})}_{T}$ > 0. Equation-4 provides a mathematical basis to rationalize the effect of PEG on FUS-buffer mixture (Fig. S8). Starting with a homogeneous FUS-buffer mixture without any crowder, the system is stable and hence $\mu_{binary}^{'}>0$. As the concentration of PEG increases, the chemical-potential derivative, ${(\frac{\partial\mu_{pro}}{\partial c_{pro}})}_{T}$ decreases and becomes zero (i.e., the spinodal condition) at a sufficiently high concentration of the crowder. Further increase in the crowder concentration leads to a spontaneous separation of the mixture into a dense and a dilute phase due to inherent thermodynamic instability (*i.e.,* ${(\frac{\partial\mu_{pro}}{\partial c_{pro}})}_{T,c_{pol}}<0$; Fig. S8). The net effect of the crowder here may be physically interpreted as an effective increase in FUS-FUS attraction, manifested by depletion interactions. In case of starting FUS concentration ≥ 2 µM, the system phase separates without any crowder and hence, $\mu_{binary}^{'}<0$ to start with. In that case, the relative magnitude of effective protein-protein attraction, and hence, the density of protein molecules in the condensed phase increases with the crowder concentration. This is experimentally verified and shown in Fig. 5d (*main-text*). Together, this theoretical argument provides a basis for our observations that polymer crowders enhance FUS LLPS and condensed phase material properties.

In summary, the presented thermodynamic model provides a theoretical rationale of our experimental observations. We note that the current model is simplified and representative of ideal polymer crowders in dilute regime. Several factors including crowder-RNP interactions, polymer/RNP chain entanglement, etc. should be carefully considered to develop this model further. One exciting future direction would be to study how the RNP-RNP interactions are scaled with a crowder concentration for individual RNPs with distinct LCD sequences, which we envision will be dependent on the LCD sequence itself.

**References:**

1 Choi, K. J. *et al.* A Chemical Chaperone Decouples TDP-43 Disordered Domain Phase Separation from Fibrillation. *Biochemistry* **57**, 6822-6826, doi:10.1021/acs.biochem.8b01051 (2018).

2 Wang, Y. & Annunziata, O. Comparison between protein-polyethylene glycol (PEG) interactions and the effect of PEG on protein-protein interactions using the liquid-liquid phase transition. *J Phys Chem B* **111**, 1222-1230, doi:10.1021/jp065608u (2007).

3 Lekkerkerker, H. N. W., Poon, W. C. K., Pusey, P. N., Stroobants, A. & Warren, P. B. Phase Behaviour of Colloid + Polymer Mixtures. *EPL (Europhysics Letters)* **20**, 559 (1992).

4 Annunziata, O. *et al.* Effect of polyethylene glycol on the liquid-liquid phase transition in aqueous protein solutions. *Proc Natl Acad Sci U S A* **99**, 14165-14170, doi:10.1073/pnas.212507199212507199 (2002).
